# bettercallsal: better calling of *Salmonella* serotypes from enrichment cultures using shotgun metagenomic profiling and its application in an outbreak setting

**DOI:** 10.3389/fmicb.2023.1200983

**Published:** 2023-08-02

**Authors:** Kranti Konganti, Elizabeth Reed, Mark Mammel, Tunc Kayikcioglu, Rachel Binet, Karen Jarvis, Christina M. Ferreira, Rebecca L. Bell, Jie Zheng, Amanda M. Windsor, Andrea Ottesen, Christopher J. Grim, Padmini Ramachandran

**Affiliations:** ^1^Center for Food Safety and Applied Nutrition, U.S. Food and Drug Administration, College Park, MD, United States; ^2^Center for Veterinary Medicine, U.S. Food and Drug Administration, Laurel, MD, United States

**Keywords:** metagenomics, *Salmonella*, BAM, quasi-metagenomics, foodborne

## Abstract

Most current *Salmonella* subtyping analyses rely on whole genome sequencing (WGS), which focuses on the high-resolution analysis of single genomes or multiple single genomes from the isolated colonies on microbiological agar plates. In this study, we introduce bioinformatics innovations for a metagenomic outbreak response workflow that accurately identifies multiple *Salmonella* serovars at the same time. bettercallsal is one of the first analysis tools to identify multiple *Salmonella enterica* serotypes from metagenomic or quasi-metagenomic datasets with high accuracy, allowing these isolate-independent methods to be incorporated into surveillance and root cause investigations. It was tested on an *in silico* benchmark dataset comprising 29 unique *Salmonella* serovars, 46 non-*Salmonella* bacterial genomes, and 10 viral genomes at varying read depths and on previously well-characterized and sequenced non-selective primary and selective enrichments of papaya and peach samples from separate outbreak investigations that resulted in the identification of multiple *Salmonella* serovars using traditional isolate culturing and WGS as well as nucleic acid assays. Analyses were also conducted on these datasets using a custom-built *k-mer* tool, SeqSero2, and Kallisto to compare serotype calling to bettercallsal. The *in silico* dataset analyzed with bettercallsal achieved the maximum precision, recall, and accuracy of 100, 83, and 94%, respectively. In the papaya outbreak samples, bettercallsal identified the presence of multiple serovars in agreement with the Luminex^®^ xMAP assay results and also identified more serovars per sample, as evidenced by NCBI SNP clustering. In peach outbreak samples, bettercallsal identified two serovars in concordance with *k*-mer analysis and the Luminex xMAP assay. The genome hit reported by bettercallsal clustered with the chicken isolate genome, as reported by the FDA peach outbreak investigation from sequenced isolates (WGS). Overall, bettercallsal outperformed *k-mer*, Seqsero2, and Kallisto in identifying multiple serovars from enrichment cultures using shotgun metagenomic sequencing.

## Introduction

In the United States, *Salmonella* is a leading bacterial cause of foodborne outbreaks. Therefore, precise and rapid identification of *Salmonella* serotypes from suspect food matrices is critical for successful source attribution of illness outbreaks (Scallan et al., 2011). From 2000 to 2020, 2.85% of U.S. foodborne *Salmonella* outbreaks were attributed to multiple S*almonella* serotypes (CDC, 2022). Recent foodborne outbreaks that have been attributed to multiple *Salmonella* serotypes force us to question whether these are rare events or whether previous methods did not have the resolution to provide an accurate picture of the complex ecology that is associated with outbreak etiologies (Hassan et al., 2019; FDA, 2021; Whitney et al., 2021).

Serotyping has been at the core of public health monitoring of *Salmonella* infections for over 50 years. Since the 1960's, public health scientists in the United States have used serotyping to identify, subtype, and track *Salmonella* outbreak strains to their sources. The U.S. Centers for Disease Control and Prevention (CDC) has published an atlas of *Salmonella* in the United States featuring 32 serotypes of *Salmonella* commonly associated with clinical illness (CDC, 2020), thus highlighting the importance of precise identification and clustering of *Salmonella* serotypes. Current surveillance for *Salmonella* is generally limited to the detection of only the most abundant serotype(s) in a sample due to biases in culture-based screening approaches. Thus, some serotypes that are present in low abundance in an enrichment culture may remain undetected and, in some cases, even the etiological agent causing the outbreak may remain undetected despite epidemiologic links to the food being tested (Harvey and Price, 1967; Singer et al., 2009). Next-generation sequencing (NGS) technologies have ushered in an era of precision analysis, transforming the way we detect, identify, and conduct source tracking of foodborne pathogens (Rantsiou et al., 2018; Unno et al., 2018). As we continue to discover more information about outbreak etiology, the potential of quasi-metagenomic (sequencing from enrichments) methods for rapid detection is becoming abundantly clear (Ottesen et al., 2016, 2020).

Accurate subtyping and subsequent clustering of *Salmonella* serotypes associated with a foodborne outbreak event is essential for successful investigation and traceback to a specific food or an environmental source. Most metagenomic profiling tools that use either marker- or *k-mer*-based approaches for classification are sensitive down to the species rank (McIntyre et al., 2017) and cannot accurately discern between highly clonal *Salmonella* spp. serotypes. Assembly-based approaches have also been widely used in metagenomics, especially to obtain cluster information for traceback (Buytaers et al., 2021). The recent advent of DNA sketching-based algorithms has enabled much more efficient and accurate processing of large amounts of data, with the potential to analyze the sequencing data in “real-time” (Rowe, 2019). In the case of quasi-metagenomic datasets, we have successfully used the CFSAN SNP pipeline for clustering and traceback in the past (Ottesen et al., 2020), but the pipeline performs best when there is appreciable coverage at all possible sites and when there is a single etiologic agent (Davis et al., 2015). These criteria are difficult to achieve in every quasi-metagenomic dataset and are highly dependent on the food matrix in terms of the relative abundance of the pathogen of interest and the burden of the microbial community as a whole. Using approaches like *k-mer* or assembly-based methods or adapting tools that are applicable to whole genome sequencing in a multi-serovar outbreak did not result in the detection of all the serotypes and the clustering information for a traceback. We built bettercallsal primarily based on DNA sketching algorithms (Ondov et al., 2019; Pierce et al., 2019) to address the need to identify multiple *Salmonella* spp. serotypes from metagenomic or quasi-metagenomic datasets. We leverage the NCBI Pathogen Detection (PD) project (Sayers et al., 2021) and provide hyperlinks to isolate genome(s) hits via the NCBI Isolates Browser, which, in turn, allows visualization within the NCBI SNP Tree Viewer if the genome hit was a member of a clonally related cluster (Sayers et al., 2021).

This new *Salmonella* serotyping tool for metagenomic datasets can have a true impact on understanding differences in the dynamics of co-occurring *Salmonella* serotypes and can have a significant impact on understanding the ecology of this pathogen with respect to food safety and public health measures. The bettercallsal workflow is licensed under MIT and is freely available for download and use at: https://github.com/CFSAN-Biostatistics/bettercallsal.

## Materials and methods

### Simulated datasets

Four sets of *in silico* Illumina datasets were generated with InSilicoSeq (Gourle et al., 2019) using *Salmonella* and non-*Salmonella* microbial genome assemblies with the 2x300 bp MiSeq error model. For the first three sets, the microbial community composition includes 29 *Salmonella* genomes representing unique *Salmonella* serotypes of importance in foodborne diseases, along with 46 non-*Salmonella* bacteria and 10 viral and phage species (Supplementary Table 1).

Although it is rare to notice such a high number of *Salmonella* spp. serotypes (*n* = 29) in a single sample in an outbreak setting, to test the thresholds of serotype identification with bettercallsal, simulated reads were generated for the first dataset using the genome assembly FASTA files from the microbial community composition (*n* = 29 + 46 + 10) at an equal coverage of 5X for each of the 29 unique *Salmonella* serotypes (sal-cov5x) and with a coverage of 0–12X for the rest of the genomes (Supplementary Table 1). This same composition of 85 (*n* = 29 + 46 + 10) genomes was used to generate the second read set, with the only change being that the coverage of the 29 unique *Salmonella* genomes was between 1X and 5X (sal-cov1-5x; Supplementary Table 1). A third read set was generated with a log-normal abundance distribution for all 85 genomes (sal-abn) using the same random seed (−−seed 27) at varying read depths ranging from 0.5 to 5 million read pairs (Supplementary Table 1). Finally, to mimic some of the recently identified papaya outbreak samples in which multi-serovar *Salmonella* serotypes were isolated from a single sample (Whitney et al., 2021), we generated four additional simulated Illumina paired-end datasets using InSilicoSeq (Gourle et al., 2019) with the 2x300 bp MiSeq error model. Each dataset consisted of a mixture of 3–5 unique *Salmonella enterica* serotypes along with the same 46 non-*Salmonella* bacterial and 10 viral and phage species (mix1 to mix4; Table 1, Supplementary Table 1). The *Salmonella* genomes in these mixes are closely related, with the minimum average nucleotide identity (ANI) between a pair of genomes at 98.2% and the maximum ANI at 99.4% (Supplementary Table 1).

**Table 1:** The *Salmonella spp*. serotypes in each of the mix1 to mix4 simulated datasets.

**Dataset**	**Isolate accession**	**Computed serotype**
mix1	GCA_022512175.1	Agona
	GCA_022653335.1	Anatum
	GCA_020915285.1	Javiana
	GCA_007147185.1	Newport
	GCA_019151245.1	Senftenberg
mix2	GCA_015076525.1	Duisburg
	GCA_009443175.1	Mbandaka
	GCA_016347165.1	Nottingham
	GCA_007014865.1	Oranienburg
	GCA_013764765.1	Sandiego
mix3	GCA_016580005.1	Alachua
	GCA_007626015.1	Gaminara
	GCA_007381005.1	Reading
	GCA_020956055.1	Saintpaul
mix4	GCA_023802815.1	Berta
	GCA_006632585.1	Enteritidis
	GCA_005557195.1	Gallinarum or Enteritidis

InSilicoSeq was used with −−seed 27 and 2x300 bp MiSeq and 2x150 bp NextSeq error models. The other 56 non-Salmonella genome accessions that are part of each of the mixes are described in Supplementary Table 1.

The four simulated read generation steps discussed above (sal-cov5x, sal-cov1-5x, sal-abn, and mix1 to mix4) were repeated to generate NextSeq datasets. InSilicoSeq does not provide a pre-computed error model for the Illumina NextSeq platform. To generate a NextSeq error model using InSilicoSeq, we used the 2x150 bp NextSeq 500 parmesan cheese food matrix FASTQ dataset (SRR12959987) from the METAnnotatorX2 study (Milani et al., 2021). The only difference is that, for the sal-abn dataset (third read set), the reads were generated from 0.5 to 10 million read pairs compared to 0.5–5M read pairs for MiSeq to compensate for the shorter read lengths of the NextSeq instrument.

### Database generation

A custom database was generated via the “bettercallsal_db” workflow. It automates the process of downloading the datasets from the NCBI Pathogen Detection (PD) database and preparing a list of pre-formatted database flat files by taking the Pathogen Detection Group (PDG) release identifier as input (Ex: PDG000000002.2537). Whole-genome sequencing (WGS) of each *Salmonella* isolate submitted to NCBI PD is cataloged per the metadata structure along with the *in silico* serotyping performed on the isolate assembly by SeqSero2 (Zhang et al., 2019), which is disseminated via the metadata field called “computed_serotype.” This field is used to associate the genome hits with a serotype within the main bettercallsal analysis workflow. Two database types are created with the “bettercallsal_db” workflow. The first is a collection of isolate genome FASTA files based on SNP Cluster participation (“per_snp_cluster”) of the genome, wherein the single longest contiguous genome by Scaffold N50 or Contig N50 size is retained per SNP Cluster ID. The second type of database is a collection of isolates for each “computed_serotype” (“per_computed_serotype”) based on the downloaded metadata. Up to 10 genomes are retained in the *per_computed_serotype* database based on the following “waterfall” pseudo-algorithm:

For all rows from the NCBI Pathogens metadata file for *Salmonella* where “computed_serotype” cell values are not null, i.e., for each valid “computed_serotype,” do the following:

1. Use the scaffold N50 size of each isolate to sort the metadata in a descending fashion. If the scaffold N50 size is not available, use the contig N50 size for subsequent steps.
2. While sorting, if two genomes' scaffold N50 sizes are equal for a given serotype, include both.
3. Retain up to 10 (user-configurable) isolates' metadata for each “computed_serotype.” This step has no effect when there are < 10 isolates available for a “computed_serotype.”
4. Finally, fetch the assembly FASTA from NCBI for up to 10 isolates.

For both the *per_snp_cluster* and *per_computed_serotype* databases, all relevant metadata files are also created and indexed to track accession, SNP Cluster IDs, and serotypes, which are used during the tabulation of the results. The difference between the *per_snp_cluster* and *per_computed_serotype* databases is that the first prioritizes genome collection based on SNP clusters, and therefore, not all serotypes may be represented in the database. For example, a total of 668 unique serotypes are indexed in v0.3.0 of bettercallsal in the *per_snp_cluster* database, which includes all possible foodborne serotypes, whereas ~1,824 serotypes are covered in the *per_computed_serotype* database. A MASH sketch is created for each database type (Ondov et al., 2016). By default, the bettercallsal workflow uses the *per_snp_cluster* database for two reasons: the first being that the genome FASTA collection covers all possible serotypes of relevance with respect to foodborne illnesses, and the second being that the genome assemblies are not as fragmented when compared to the *per_computed_serotype* genome collection, where most of the *antigen_formula in silico* predictions are incomplete due to fragmented genome assemblies. However, the user has the ability to switch the database type. All the bettercallsal analyses discussed herein were performed against the PDG000000002.2537 release of the NCBI Pathogen Detection Database for *Salmonella*.

### Analysis workflow

**Figure 1:**
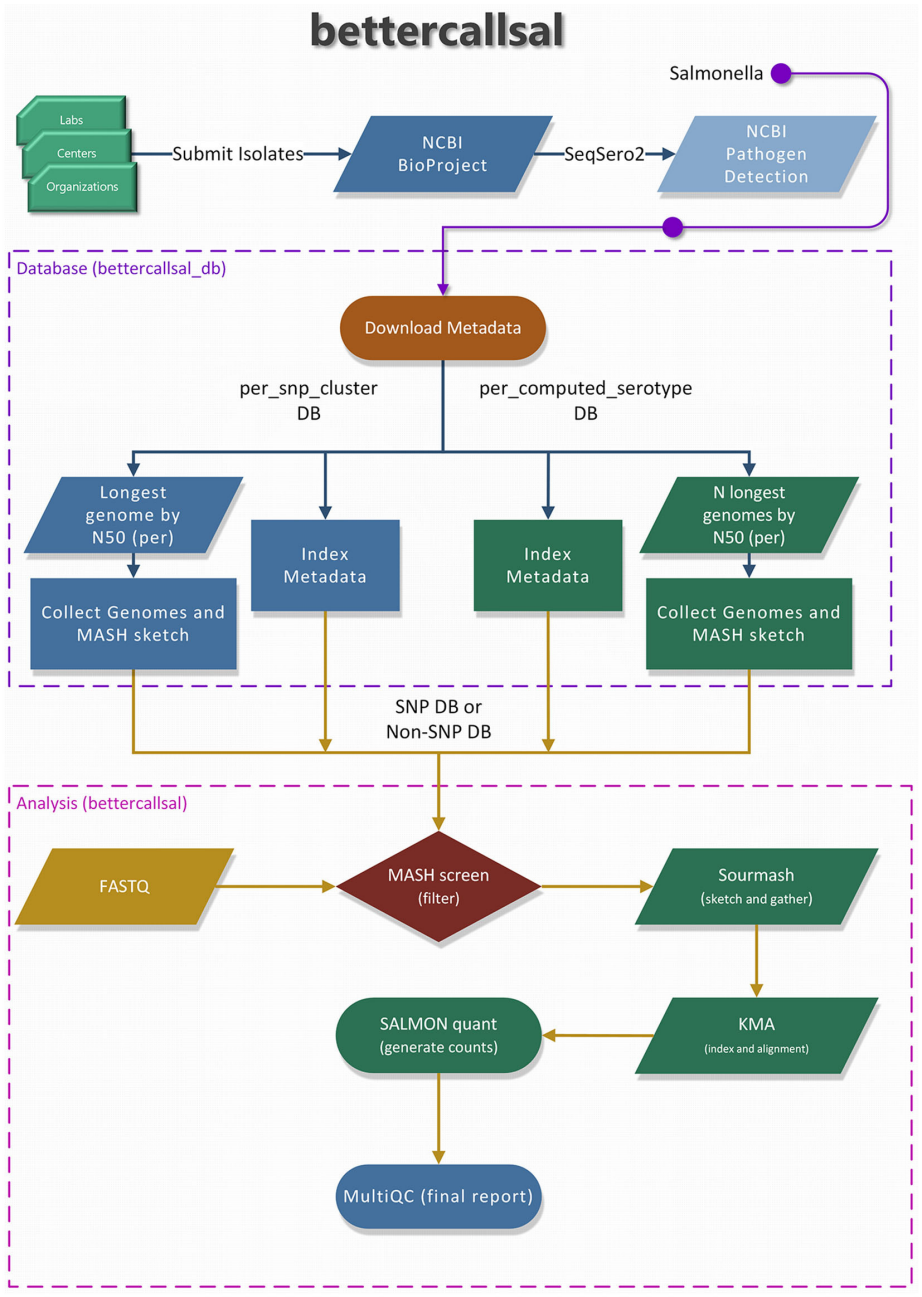
An overview of the “bettercallsal_db” and the main bettercallsal analysis workflow. First, the metadata for *Salmonella* is downloaded from the NCBI Pathogen Detection project. In the next step, all the GenBank (GCA_) and RefSeq (GCF_) accessions are used to create an accession catalog to query NCBI and to retrieve assembly statistics, such as contig N50 and scaffold N50. For the “per_snp_cluster” database, a single longest genome by N50 size is retained, and for the “per_computed_serotype” database, up to 10 longest genomes by N50 size are retained for each of the “computed_serotypes,” as discussed in Materials and methods. Finally, for both database types, the contigs are joined by 10 N's, and a MASH sketch is created. Certain pre-formatted flat files were also created and used during the main analysis workflow.

A brief overview of the bettercallsal workflow, which is written in Nextflow (Di Tommaso et al., 2017; Ewels et al., 2020), is presented in Figure 1. The main analysis workflow is a single-label metagenomic classification, wherein each genome assembly/accession match is mapped to the corresponding pre-indexed metadata. bettercallsal identifies multi-serovar populations in metagenomic and quasi-metagenomic sample datasets by first incorporating genome filtering to remove very low abundance hits, followed by read alignment and read counting to assign serotypes for a given dataset. It relies on the metadata of the *Salmonella* isolate assemblies made available by the NCBI Pathogen Detection (PD) project, which is continually updated based on the submission of new isolates from several participating U.S. public health agencies and international partners. If the input reads are paired-end, they could be merged on overlap using BBMerge (Bushnell et al., 2017).

The analysis starts with a “screen” command from MASH (Ondov et al., 2019) to generate a list of genome matches based on the fraction of bases shared between the genome sketch and the sequencing read sketch for each sample dataset, which is then sorted in descending order. Up to the top 10 unique MASH “screen” hits are used to perform additional genome fraction filtering with sourmash (Pierce et al., 2019), which is also used to generate an average nucleotide identity (ANI) containment matrix.

Subsequently, an “on-the-fly” KMA (*k-mer* alignment; Clausen et al., 2018) genome indexing and alignment is performed to further refine the genome hits. The KMA results are used to generate read counts with Salmon (Patro et al., 2017) in “−−meta” mode. The above workflow is run in parallel for all samples, the results are aggregated, and the serotype is assigned based on the “computed_serotype” column associated with each genome accession. All parameters of each tool are user-configurable via command-line options, including the threshold for filtering the top unique serotype hits after the MASH “screen” step (−−tuspy_n). A brief MultiQC (Ewels et al., 2016) report is generated in the final step of the workflow. The stand-alone MultiQC HTML report (data availability) contains multiple relevant sequence quality metrics and visualizations, an ANI matrix between samples and genomes, an aggregated results table of serotype calls with integrated hyperlinks to the NCBI PD Isolates Browser, and a Salmon read count plot showing the proportions of identified serotype(s) within each sample. Most of the visualizations are interactive, and the results tables can be downloaded. Additionally, the software version of each tool used in each of the workflow steps is reported for version control and the reproducibility of the results.

### Custom *k-mer* analyses

The *k*-mer analysis of the sequencing datasets was conducted using an in-house developed *k*-mer database. The general approach to the development of the database has been described previously (Leonard et al., 2015; Patro et al., 2016). Briefly, the bacterial composition was determined from shotgun sequencing using custom C++ programs developed to compile a *k*-mer signature database containing multiple unique 30 bp k-mer sequences per species and then identify each read in the input file using the 30 bp probes. For each bacterial species or subspecies, each non-duplicated 30-mer from a reference whole genome sequence was entered into a database. We removed all *k*-mers not found in at least two-thirds of a set of additional genome sequences from the same species, and we removed all *k*-mers found in genomes from other species. The resulting *k-mer* database used in this study contains 5,900 target entries, each consisting of ~40,000 (range 44–80,000) unique *k*-mers. The database includes 1,100 different bacterial genera and 3,500 species. Normalization was performed to correct for bias due to the differing number of *k*-*mers* used per database entry, and the results were tabulated as a percent of identified reads (contribution to the microbial population of identified species) for each database entry. Confirmation of low-level species calls was performed by BLAST analysis of the reads. Based on the hits and the number of unique *k-mers* identified per taxon per sample, a threshold was set at 1% relative abundance. Any hits to taxa with < 1% relative abundance were grouped with other genera with < 1% relative abundance.

### Comparative analyses

All analyses were performed on the CFSAN Raven2 High-Performance Computing (HPC) Cluster, where each compute node had 20 CPU cores with an Intel(R) Xeon(R) E5-2650 chipset running at 2.30 GHz and a minimum of 120 GB memory. The FASTQs generated at the time of the outbreak were currently analyzed using bettercallsal, an in-house bacterial *k*-mer approach (Patro et al., 2016), SeqSero2 (Zhang et al., 2019), and Kallisto (Bray et al., 2016).

Prior to developing bettercallsal, we attempted to repurpose the non-alignment-based RNA-seq tool, Kallisto (Bray et al., 2016), as it has been previously reported as a potential candidate for variant typing of mixed samples (Baaijens et al., 2022) and had a high accuracy in identifying SARS-Cov2 variants present in sewage-derived sample pools (Kayikcioglu et al., 2023). Kallisto indexing was performed on the H and O antigen sequences distributed by the SeqSero2 package version 1.2.1 (H_and_O_and_specific_genes.fasta) via Kallisto (version 0.48) using default parameters. To obtain abundance estimates, we classified the FASTQ files in a paired-end fashion against this index using the default parameters and parsed the plain text output for result aggregation and visualization.

Downstream data analysis and visualization of the bacterial taxonomic profiles were carried out in RStudio (v.1.3.1093) using the following R packages: ggplot2 (v3.4.1), dplyr (v1.1.0), reshape2 (1.4.4), ggh4x, and stringr (v1.5.0).

### Metrics used to evaluate the performance of bettercallsal

The accuracy and performance of bettercallsal were evaluated using the precision and recall metrics (Wood and Salzberg, 2014; McIntyre et al., 2017) on the simulated datasets. For this study, we were primarily focused on accurately assigning the *Salmonella* spp. serotype to each of the metagenomic or quasi-metagenomic samples, and thus, for the simulated datasets, the “true positives” (TP) represented the proportion of the “ground truth” *Salmonella* spp. serotypes that were expected to be identified, whereas the “false positives” (FP) represented the proportion of *Salmonella* serotypes that were incorrectly assigned to a different *Salmonella* serotype. The number of “true negatives” (TN) in this case was constant at 56, as these 46 non-*Salmonella* bacterial and 10 viral and phage genomes were not present in the “bettercallsal_db,” and thus, bettercallsal did not correctly identify any of these microbial species. Finally, “false negatives” (FN) represented the proportion of serotypes that were identified as absent (no call) when they were supposed to be present (Supplementary Table 1). The precision metric is synonymous with the positive predictive value, i.e., the ability of the workflow to identify the “ground truth” *Salmonella* spp. serotypes from a sample, whereas accuracy is a measure of the total number of correct predictions (positive or negative) over a total number of predictions. Recall, or sensitivity, evaluates the ability of bettercallsal to minimize the cases of “false negatives.”

For the custom *k-mer* method, only precision and recall metrics were calculated since the number of “true negatives” (TNs) was not constant at 56, as the database sequences included many non-*Salmonella* genera. It was also not feasible to calculate any metrics for SeqSero2 and Kallisto runs on any of the simulated datasets because SeqSero2, by design, did not call multiple serotypes in any of the simulated scenarios. For Kallisto, owing to the nature of the database composition using O-group and H1 and H2 gene sequences, the results were hits or abundances to independent O and H sequences rather than actual serotype calls, but the number of positive hits was counted based on the hits to the FASTA identifier of the O and H genes.

### Papaya outbreak samples

In 2017, the FDA investigated a multistate outbreak involving Maradol papayas (Whitney et al., 2021). A total of 15 papaya fruits from Farm A and Farm C were analyzed for *Salmonella* using the following metagenomic methods. Farm A papaya fruits were all aerobically enriched in modified buffer peptone water [mBPW FDA Bacteriological Analytical Manual (BAM) broth M192b] for 24 h at 35°C and then transferred to Rappaport-Vassiliadis (RV; BAM broth M132), tetrathionate (TT; BAM broth M145) broths for selective enrichment. Selective enrichment of Farm A papayas was analyzed using shotgun metagenomic profiling. Farm C papayas were aerobically pre-enriched in mBPW at 35°C or anaerobically at 42°C in tryptone broth (BAM medium M136), supplemented with 5 mM glutathione and 0.35 mM tetrathionate, followed by aerobic selective enrichment in RV at 42°C and modified tetrathionate (mTT; TT lacking brilliant green with 1% I_2_ KI) broth at 43°C for 24 h. DNA was extracted using the Qiagen DNeasy Blood and Tissue Kit according to the manufacturer's instructions for pre-enrichment broth and selective enrichment broth. Shotgun metagenomic profiling was performed on culture enrichments as described below.

All selective enrichments were also plated on xylose-lysine deoxycholate (XLD), Hektoen enteric agar (HE), and bismuth sulfite (BS) agars for *Salmonella* isolation. Presumptive-positive *Salmonella* was re-streaked on trypticase soy agar (TSA) and further confirmed on a Vitek MS microbial identification system (bioMérieux, Durham, NC, USA). Confirmed *Salmonella* isolates from selected Farm C samples were serotyped using the Luminex xMAP *Salmonella* Serotyping Assay (Luminex, Madison, WI, USA). Briefly, DNA was extracted from 20 confirmed isolates per sample using the Bio-Rad InstaGene matrix (Bio-Rad, Hercules, CA, USA), and serotype identification were determined following previously published protocols (Fitzgerald et al., 2007; McQuiston et al., 2011).

### Peach outbreak samples

In 2020, the FDA investigated an outbreak of *Salmonella* Enteritidis infections linked to the consumption of peaches (FDA, 2021). Peach fruits and leaves from an implicated field were weighed and combined with a universal pre-enrichment broth (UPB; BAM broth M188) at a ratio of 1:9 (w:v), or more to fully submerge the leaves. Fruit and leaves were sonicated with an output setting of 112 W for 60 s at room temperature. There is not a validated BAM method for tree leaves or peaches with a sonication step, so a green fluorescent protein (GFP)-tagged strain of *S*. Gaminara (GPF SAL 5695) was inoculated into one peach and one leaf matrix sample at an inoculum level of 30 cells or less per sample as a process and matrix control. After sonication, the broth was aseptically transferred to a new Whirl-Pak^®^ bag and incubated overnight at 35°C. RV and TT media were inoculated and incubated at 42°C for 24 h. All selective enrichments were plated on xylose-lysine-tergitol 4 (XLT-4), Hektoen enteric agar with 5 ug/ml novobiocin (HE+N), and BS agars for *Salmonella* isolation. DNA extraction from primary enrichments and selective enrichments was conducted using the Promega Maxwell^®^ RSC Cultured Cells DNA Kit (Promega, WI, USA, AS1620) according to the manufacturer's specifications on the Promega Maxwell^®^ RSC 48 instrument. Shotgun metagenomic profiling was performed on the DNA extracted from the selective enrichments as described below.

### Sequencing library preparation

Quasi-metagenomic DNA libraries were prepared for papaya sample enrichment during the 2017 outbreak investigation using the Nextera XT Library Prep according to the manufacturer's specifications (Illumina, CA, USA). Sequencing was performed on the NextSeq 550 system with 2 × 150 cycles using the NextSeq 500/550 v2.5 High Output Kit (150 cycles). Libraries were diluted to 1.8 pM according to the manufacturer's specifications (NextSeq Denature and Dilute Libraries Guide). Papaya samples were sequenced at a minimum and maximum read depths of 1.9 million and 46 million paired-end reads, respectively.

Quasi-metagenomic DNA libraries were prepared during the 2020 outbreak investigation for peach sample enrichment using the Illumina DNA prep method according to the manufacturer's specifications (Illumina, CA, USA). Sequencing was performed on the NextSeq 550 system at 2 × 150 cycles using the NextSeq 500/550 v2.5 High Output Kit (150 Cycles). Libraries were diluted to 1.8 pM according to the manufacturer's specifications (NextSeq Denature and Dilute Libraries Guide). The minimum read depth achieved for the peach datasets was 12 million paired-end reads, and the maximum read depth was 40 million paired-end reads.

### Data availability

All data are publicly available at NCBI associated with BioProject PRJNA952520. The download URLs for the simulated Illumina reads are provided in Supplementary Table 1. The final MultiQC HTML reports generated for the papaya and peach outbreaks discussed in this study are available at: https://research.foodsafetyrisk.org/bettercallsal/manuscript/papaya_outbreak_results.html and https://research.foodsafetyrisk.org/bettercallsal/manuscript/peach_outbreak_results.html, respectively.

## Results

### bettercallsal consistently assigns the correct serotype in simulated datasets

#### MiSeq simulated reads

**Figure 2:**
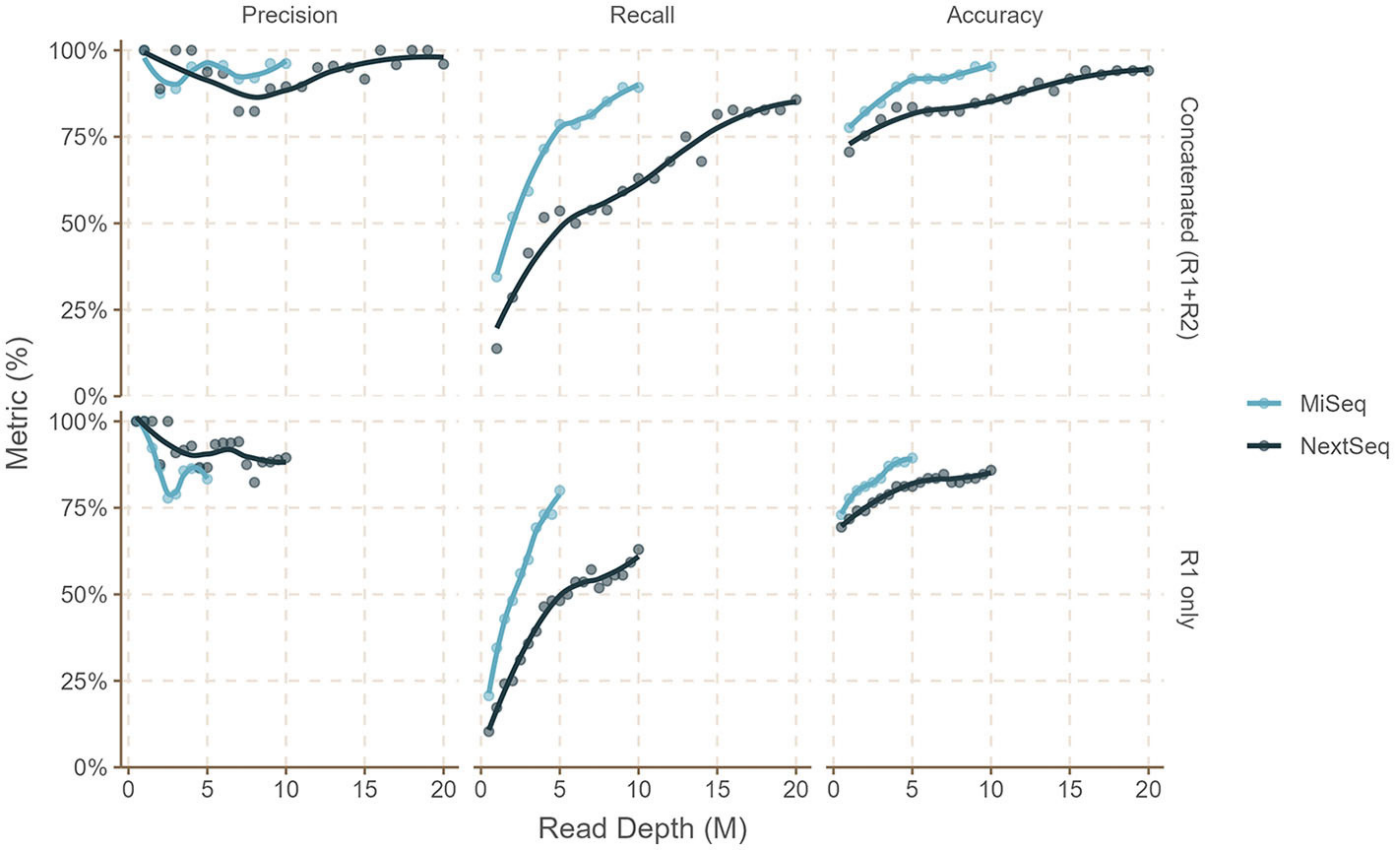
Performance of the bettercallsal workflow on the sal-abn simulated dataset shows that precision, recall, and accuracy increase with increasing read depth. Beyond 9–10 million read depth (R1+R2), there are diminishing returns for MiSeq (2x300 bp) reads, while similar or better performance is achieved between 16 and 20 million read depth (R1+R2) for NextSeq (2x150 bp) reads. The maximum precision, recall, and accuracy achieved were 96.1, 89.2, and 95.2% for 9M (R1 + R2) and 10M (R1 + R2) MiSeq reads compared to 100, 82.7, and 94.1% for 16M (R1+R2) NextSeq reads.

**Figure 3:**
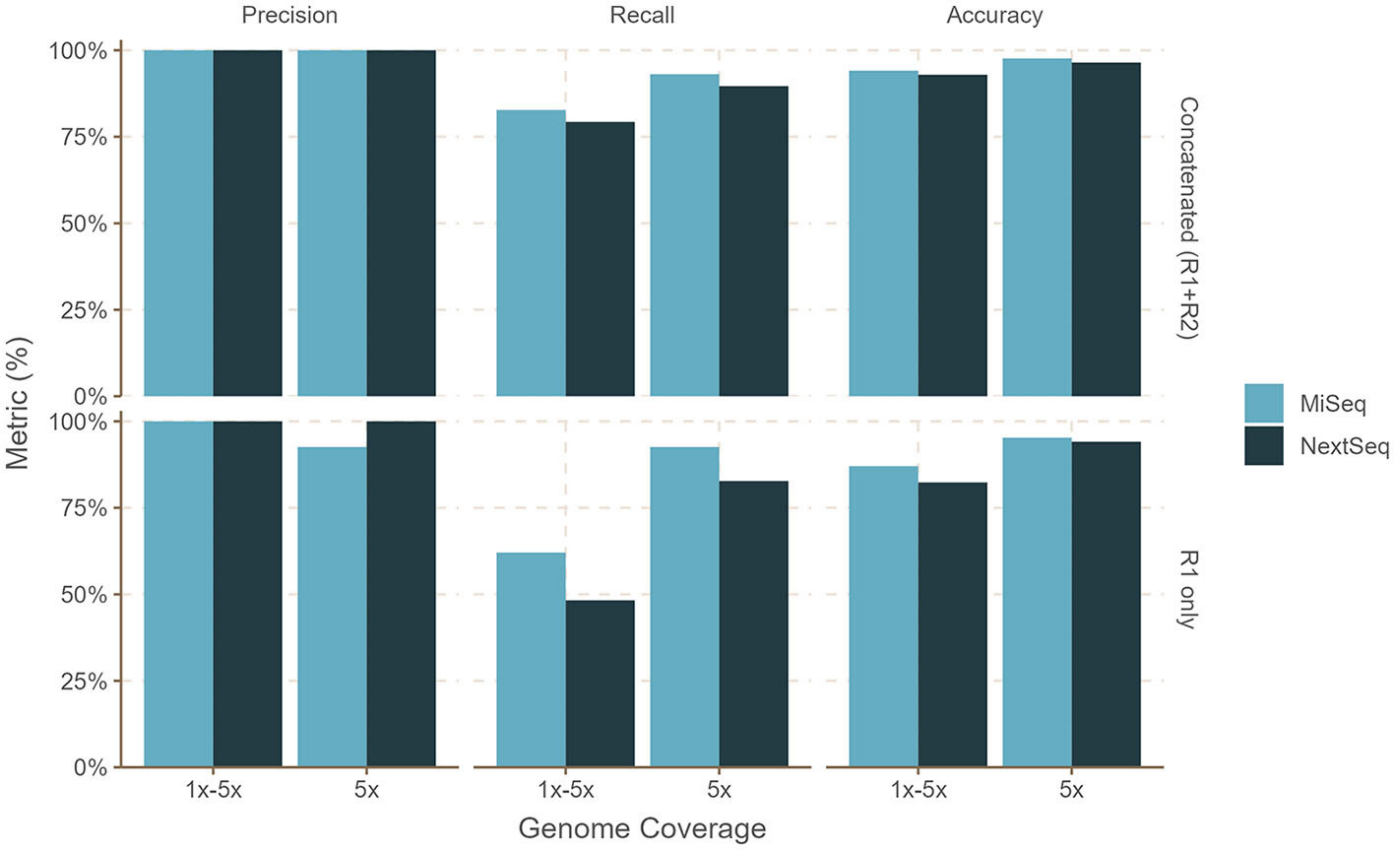
Performance of bettercallsal on simulated datasets with MiSeq (2x300 bp) or NextSeq (2x150 bp) error models. *Salmonella* genomes (*n* = 29) were generated with an equal 5x coverage (sal-cov5x) and an unequal, i.e., between 1 and 5x coverage (sal-cov1-5x), along with bacterial and viral genomes (*n* = 46 + 10), which were generated with coverage between 1 and 12x. The maximum precision, recall, and accuracy of 100, 93, and 97% were achieved for the sal-cov5x MiSeq dataset compared to 100, 89.6, and 96.4% for the sal-cov5x NextSeq dataset. For the sal-cov1-5x MiSeq dataset, the maximum precision, recall, and accuracy was 100, 82.7, and 94.1% compared to 100, 79.3, and 92.1%, for the sal-cov1-5x NextSeq dataset.

*In silico* dataset analyses revealed that precision, recall, and accuracy increase with increased read depth, and beyond the depth of 5 million read pairs for MiSeq (2x300 bp) and 10 million read pairs for NextSeq (2x150 bp), diminishing returns were observed (Figures 2, 3, Supplementary Table 1) in all of the simulated read set scenarios (sal-abn, sal-cov5x, sal-cov1-5x, and mix1 to mix4). When we attempted bettercallsal on paired-end datasets by merging the read pairs on overlap, we observed that these merged datasets performed poorly when compared to single-end or concatenated (R1+R2) datasets because only ~25–50% of read pairs were merged successfully on overlap. We suspect that this is due to the stochastic nature of the insert size in paired-end data causing read information loss during merging (Sahlin et al., 2015), whereas we observed that concatenating the R1 and R2 paired-end sequencing files and running bettercallsal data yielded superior results.

For the sal-abn dataset, which was generated using InSilicoSeq (Gourle et al., 2019) with a log-normal abundance distribution, bettercallsal achieved precision, recall, and accuracy of 83, 80, and 89% for single-end reads at 5 million read depth (R1), whereas precision, recall, and accuracy of 100, 93, and 98% were observed for the concatenated (R1+R2) reads at 10 million read depth (Figure 2, Supplementary Table 1). For the sal-cov5x dataset, we observed the maximum precision, recall, and accuracy of 100, 93, and 98% for R1+R2 reads compared to that of 100, 83, and 94% for the sal-cov1-5x dataset (Figure 3, Supplementary Table 1).

The mix1 to mix4 simulated datasets contained 3–5 unique *Salmonella* spp. serotypes and the rest of the non-*Salmonella* microbial genome assemblies (*n* = 46 + 10; Materials and methods, Table 1, and Supplementary Table 1). Here, bettercallsal achieved a maximum precision and recall of 100% at 10 million read depths (R1+R2) in all mixes except for mix2, where the maximum recall was 80%. In the mix2 dataset, bettercallsal failed to consistently identify *Salmonella* serotype Sandiego at 0.0045 relative abundance at any read depth (Supplementary Table 1).

#### NextSeq simulated reads

bettercallsal performed similarly or better on NextSeq simulated datasets, albeit at a higher read depth. This can be attributed to the shorter read lengths due to the sequencing chemistry used on the NextSeq instruments. For the sal-abn dataset, the maximum observed precision, recall, and accuracy were 100, 83, and 94%, respectively, at a read depth of 16 million (R1+R2), compared to 96, 80, and 95% at a read depth of 10 million (R1+R2) for MiSeq (Figure 2, Supplementary Table 1). For the sal-cov5x and sal-cov1-5x datasets, precision, recall, and accuracy were 100, 80, 93%, and 100, 90, and 96%, respectively (Figure 3, Supplementary Table 1). In the case of the simulated mix1 to mix4 datasets, similar to MiSeq, we observed that bettercallsal failed to identify the serotype *Salmonella* Sandiego in the mix2 dataset, resulting in a maximum precision, recall, and accuracy of 100, 88, and 98%, respectively, for this dataset (Supplementary Table 1).

### Evaluating bettercallsal against custom-built *k-mer* tool, SeqSero2, and Kallisto

The simulated run analyses revealed that bettercallsal performs consistently in calling *Salmonella* serotypes across multiple Illumina sequencing platforms. For the custom *k-mer* analyses, we used the relative abundance cutoff of >1% to gather hits for each input dataset in a paired-end mode based on our previous study (Leonard et al., 2015; Patro et al., 2016) and aggregated the results across all simulated Illumina read sets (Supplementary Table 2). bettercallsal outperformed the custom *k-mer* analysis method in all of the simulated scenarios (sal-abn, sal-cov5x, sal-cov1-5x, and mix1 to mix4), where the later achieved the highest precision and recall of 88 and 72%, respectively (Table 2, Supplementary Table 2). When SeqSero2 was run in the paired-end mode on all simulated datasets, it was reported as detecting the co-existence of multiple serotypes and generating an antigen allele FASTA, but it only called the antigen formula based on the abundance of O, H1, and H2 hits. Similarly, when considering Kallisto's reported estimated abundances >0 for the sal-abn, sal-cov5x, and sal-cov1-5x datasets, it correctly identified 22 out of 29 simulated *Salmonella* spp. serotypes in both MiSeq and NextSeq datasets compared to 28 out of 29 for bettercallsal, whereas in the mix1 to mix4 datasets, it failed to correctly identify all of the simulated serotypes in mix 2 (*n* = 3/5) and mix4 (*n* = 2/2) except mix1 and mix4 (Supplementary Table 2).

**Table 2:** Comparison of maximum achieved precision and recall on simulated reads with 29 *Salmonella* serotypes (sal-abn, sal-cov5x, and sal-cov1-5x) and various mixes (mix 1–mix 4) between bettercallsal and the custom *k-mer* method.

**Dataset**	**bettercallsal**	**Custom *k*-mer**
sal-abn-MS	96.1%, 89.2%	87.5%, 72.4%
sal-abn-NS	100%, 82.7%	87.5%, 72.4%
sal-cov5x-MS	100%, 93.1%	87.5%, 72.4%
sal-cov5x-NS	100%, 89.6%	84.5%, 72.4%
sal-cov1-5x-MS	100%, 93.1%	87.5%, 72.4%
sal-cov1-5x-NS	100%, 79.3%	84.5%, 72.4%
mix1 to mix4-MS	100%, 100%	35.7%, 100%
mix1 to mix4-NS	100%, 100%	38.4%, 100%

The Illumina MiSeq simulated dataset is noted with the -MS suffix, while the NextSeq dataset is noted with the -NS suffix.

### bettercallsal identifies multi-serovar mixtures in outbreak samples

#### Papaya outbreak

During the 2017 papaya outbreak, the FDA Office of Coordinated Outbreak Response and Evaluation team initially reported *Salmonella enterica* serotypes Agona, Gaminara, Infantis, Kiambu, Newport, Thompson, and Senftenberg associated with papayas as determined by regulatory microbiological testing, from the same two farm sources (Farms A and C), which we evaluated using metagenomic sequencing (Hassan et al., 2019; Whitney et al., 2021). Five *Salmonella* serotypes (Agona, Kiambu, Gaminara, Senftenberg, and Thompson) were identified in these regulatory samples of papayas originating from Farm A (Whitney et al., 2021). For Farm C papaya samples, FDA regulatory sampling identified *Salmonella enterica* serotypes Newport and Infantis. The particular strain of *S*. Newport is very rare and was last observed in PulseNet in 2006, and the isolated strain of *S*. Infantis was new to the database and had not been reported prior to the summer of 2017 (Hassan et al., 2019). These regulatory results serve as our “ground truth” for the evaluation of bettercallsal and comparative methods.

In the quasi-metagenomic profiles of the Farm A RV and TT selective enrichments, bettercallsal was able to identify all five serotypes identified in the initial investigation. At least one and up to four serotypes in a single sample were identified with bettercallsal in six of seven papayas (samples 2, 3, 7, 15), and no serotype was called in sample 1 (Table 3). In sample 7, bettercallsal identified an additional serotype, *S*. *enterica* ser. Barranquilla, which was not identified by Hassan et al. (2019) or Whitney et al. (2021), but in the NCBI Pathogen Detection SNP Tree Viewer, the sequence with serotype Barranquilla in our sample was closest to, clusters with sequences either deposited as or having the SeqSero2 “computed_serotype” S. Gaminara.

**Table 3:** *Salmonella enterica* serovars detected in papaya outbreak samples from Farm A by bettercallsal, xMAP, custom *k*-mer, SeqSero2, and Kallisto analyses.

**Papaya farm A sample#**	**bettercallsal**	***k*-mer**	**SeqSero2**	**Kallisto**	**xMAP**
1	No call	Senftenberg	No call	No call	Agona
2	Agona, Gaminara, Thompson, Senftenberg	Agona, Senftenberg	No call	Agona, Thompson	Senftenberg, Thompson
3	Agona, Kiambu, Senftenberg	Agona, Senftenberg	No call	Agona	Agona, Senftenberg
7	Barranquilla^*^, Gaminara, Senftenberg	Senftenberg	No call	Gaminara	Senftenberg
9	Senftenberg	Senftenberg	Senftenberg	Senftenberg	Senftenberg
14	Gaminara	No call	No call	Gaminara	Kiambu
15	Agona, Kiambu, Senftenberg	Agona, Senftenberg	Kiambu	Agona	Senftenberg

Luminex xMAP calls represent culture ground truth, but bettercallsal identified additional serotypes such as Agona, Gaminara, and Kiambu that were detected in samples 2, 7, and 15 when compared to xMAP.

^*^In the NCBI Pathogen Detector SNP Tree Viewer, the genome with serotype Barranquilla that our sample is closest to, clusters with isolates either deposited as or with the SeqSero “computed_serotype” S. Gaminara.

In comparison, the Luminex xMAP assay identified four of the five serotypes identified in the regulatory investigation and multiple serotypes (*S*. Thompson and *S*. Senftenberg) in samples 2 and 3 (Table 3). The custom *k*-mer analysis identified serotypes Agona and Senftenberg in samples 2, 3, and 15 and only Senftenberg in samples 1, 7, and 9 (Table 3). SeqSero2 identified two serotypes*, S*. Senftenberg in sample 9 and *S*. Kiambu in sample 15. Kallisto identified the serotypes Agona in samples 2, 3, and 15 and Senftenberg in sample 9. *S*. Gaminara was identified in samples 7 and 14, and *S*. Thompson was identified in sample 2 (Table 3, Supplementary Table 2). Kallisto also identified several other serotypes that are false positives (Supplementary Table 2).

In the Farm C pre-enrichment and selective enrichment, bettercallsal identified a single serotype per sample in five out of eight samples. The serotypes identified, S. Newport (samples 5, 7, 11, and 13) and S. Infantis (sample 6; Table 4), agree with the findings of the regulatory investigation, and even the genome assembly hit reported most closely matched the WGS assembly of the isolate from the regulatory outbreak investigation, as evidenced by SNP clustering to the NCBI PD.

**Table 4:** *Salmonella enterica* serovars detected in papaya outbreak samples from farm C by bettercallsal, xMAP, *k-mer*, Kallisto, and SeqSero2 analyses.

**Papaya farm C sample#**	**bettercallsal**	***k*-mer**	**SeqSero2**	**Kallisto**	**xMAP**
2	No call	No call	No call	No call	No call
5	Newport	Newport	Infantis	Newport	Infantis, Newport
6	Infantis	Infantis	Infantis	Infantis	Infantis
7	Newport	Newport	Newport	Newport	*Salmonella* group
10	No call	No call	No call	No call	No call
11	Newport	Newport	No call	Newport	*Salmonella* group
12	No call	No call	No call	No call	No call
13	Newport	Newport	No call	Newport	Newport

*k*-mer analyses and Kallisto analyses also identified several other false-positive Salmonella serotypes. Luminex xMAP calls represent the ground truth of the culture.

The Luminex xMAP assay identified the serotype *S*. Newport in samples 5 and 13 and serotype *S*. Infantis in samples 5 and 6. Samples 7 and 11 were identified as *Salmonella* only (Table 4). The custom *k*-mer analysis identified *S. Newport* in samples 5, 7, 11, and 13 and *S*. Infantis in sample 6 (Table 4). The *k-*mer analysis also identified *Salmonella* or *Salmonella enterica* in samples 5, 6, 7, 11, and 13 (Table 4). SeqSero2 identified *S*. Infantis in samples 5 and 6 and *S*. Newport in sample 7 (Table 4). Kallisto identified serotype Newport in samples 5, 7, 11, and 13 (Table 4, Supplementary Table 2). Kallisto also identified serotype Infantis in sample 6. None of the methods detected *Salmonella* in samples 2, 10, and 12 from Farm C.

#### Peach outbreak

**Figure 4:**
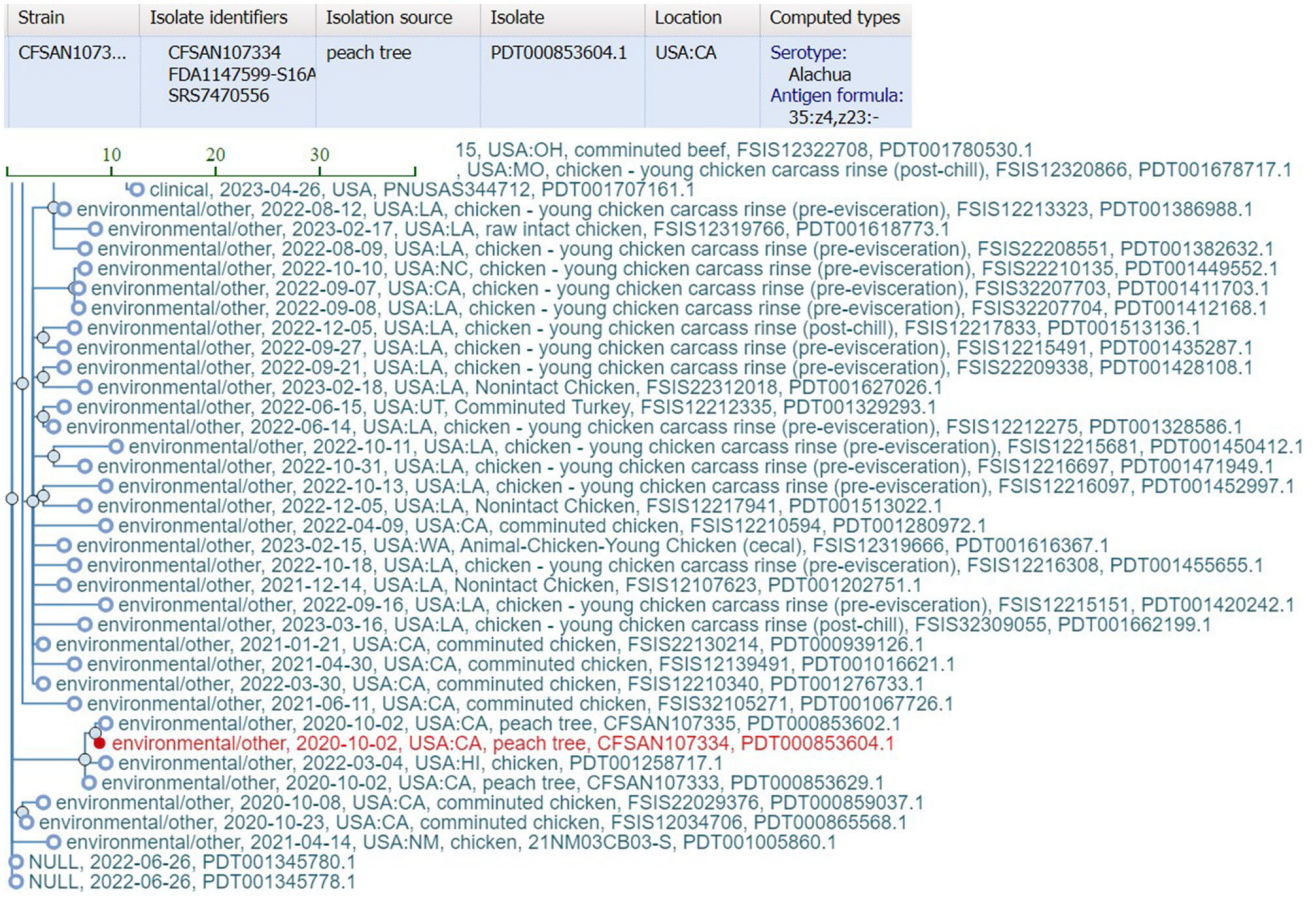
SNP cluster information and computed serotype information for the peach outbreak via external link from the bettercallsal result table of the HTML report file to the NCBI pathogen detection website. The closest genome hit (red) reported by bettercallsal clustered with the same chicken isolate genome reported in the FDA investigation of the peach outbreak (FDA, 2021).

Based on the historical outbreak data, this multistate outbreak involving peaches appears to represent a novel commodity/pathogen pair. As noted in the Methods section, some samples were spiked with GFP-labeled *S*. Gaminara, and regulatory investigation samples identified *S*. Alachua linked to isolates from poultry collected in 2019 and 2020 (FDA, 2021).

bettercallsal identified *S*. Gaminara in all GFP-labeled Gaminara-spiked enrichments of peach samples (Table 5). The serotypes Alachua and Gaminara were identified in the GFP-labeled Gaminara-spiked leaf samples. bettercallsal identified the serotype Alachua in all enrichments of the un-spiked peach and leaf samples. The genome hit reported by bettercallsal clustered with the genome of the chicken isolates reported by the FDA investigation (FDA, 2021) of the peach outbreak (Figure 4).

**Table 5:** *Salmonella enterica* serovars detected in a peach outbreak by bettercallsal, custom *k*-mer, and SeqSero2 analyses.

**Sample**		**bettercallsal**	***k*-mer**	**SeqSero2**	**Kallisto**
Spiked	Peaches	Gaminara	Gaminara	Gaminara	No call
	Leaves	Alachua, Gaminara	Gaminara	Gaminara	Gaminara
Un-spiked	Peaches	Alachua	Alachua	Alachua	No call
	Leaves	Alachua	Alachua	No Call	No call

Pre- and selective enrichment *k*-mer analysis of peaches and leaves identified the spiked GFP-labeled *S*. Gaminara and *S*. *enterica* serotype Alachua (Table 5, Supplementary Table 2). SeqSero2 identified *S*. Gaminara in both spiked peaches and leaves and *S*. Alachua in un-spiked peaches (Table 5). Kallisto abundance analysis on this sample set correctly identified the O and H (*fliC* and *fljB*) antigen profiles and identified *S*. Gaminara in the leaves (Table 5, Supplementary Table 2). However, these antigen identifications were not able to reconstruct the antigenic formula of the expected serovar(s).

### Workflow output

The bettercallsal pipeline runs primarily on the command line on UNIX-based machines. The pipeline takes as input a POSIX (or full) path to a folder containing FASTQ files. Successful execution of the workflow produces various output files, but, in general, each process within the workflow produces its own output, which is stored inside a folder named after the process name. The workflow generates a brief MultiQC (Ewels et al., 2016) HTML report at the end, which shows a table of results, with each row representing a sample and each column representing an identified serotype (Data availability). For each sample where no serotype is identified, a “−” value is used. The HTML report also displays a horizontal bar plot of read counts per “serotype” (Data availability). All the result tables and plots from the HTML report can be easily exported and saved (see the Data availability section for hyperlinks to MultiQC papaya and peach outbreak HTML reports).

### Computational resource requirements

Written in Nextflow, the bettercallsal and “bettercallsal_db” workflows readily provide inherent advantages such as process parallelization and process retry on failure. The database workflow (“bettercallsal_db”) finishes in approximately an hour with a minimum required memory of 16 GB and 8 CPU cores. Since the penultimate step of the workflow generates more than 200 individual genome scaffolding jobs, it is recommended to run the database workflow in a grid computing infrastructure or a similar setting where many processes can be queued up in parallel for execution.

The main bettercallsal workflow requires a minimum of 10 CPU cores and 16 GB to run all the workflow steps successfully. The minimum CPU core requirements can be easily modified by users by adjusting the −−max_cpus parameter. It would not be a fair comparison to evaluate Nextflow-based workflows with other established serotyping tools, such as SeqSero2, in terms of run times and memory, as the approach of setting up individual analysis steps in Nextflow workflows follows a completely different data flow philosophy. Nevertheless, Table 6 shows a bird's-eye view of the run time comparison with SeqSero2. All SeqSero2 analyses were run using 10 CPU cores. Both bettercallsal and SeqSero2 consume similar amounts of memory on all tested datasets. The increase in memory consumption with bettercallsal on the papaya outbreak datasets can be attributed to the FASTQC process, with the other main processes, such as kma, sourmash, and salmon, consuming between 1 and 2 GB (Supplementary Table 1). Gathering the run time for each of the input FASTQ datasets with bettercallsal will not be accurate as some variables are involved while running the Nextflow workflows, including the availability of the computational resources in a grid computing environment. However, bettercallsal completed the run in ~40 min when tested on all the simulated datasets as input, while SeqSero2, on average, took 6–7 min per sample (Table 6). On real outbreak datasets, such as the papaya and peach outbreak data, bettercallsal took 55 and 101 min, respectively, for all sample datasets (38 for papaya and 12 for peach), and SeqSero2 took an average of 5 min and 19 min per sample dataset, respectively (Supplementary Table 1).

**Table 6:** Run time comparison of the bettercallsal workflow with SeqSero2 in minutes, using 10 CPU cores for each run.

**mix1 to mix4 (simulated)**			
**Sample**	**bettercallsal**	**SeqSero2 (sum)**	**SeqSero2 (avg)**
sal-mix1 (3)	40	22.34	7.44
sal-mix2 (3)	40	22.31	7.43
sal-mix3 (3)	42	23.1	7.36
sal-mix4 (3)	42	20.02	6.67
**Peach outbreak**			
All samples (12)	101	291.91	19.46
**Papaya outbreak**			
All samples (38)	55	221.43	5.82

Each of the mix samples had three FASTQ input files for bettercallsal (R1+R2), whereas, for SeqSero2, the input was three read pairs. The SeqSero2 jobs were all run in parallel for each sample on the CFSAN HPC Raven2 cluster, and the “avg” column represents the actual time for all computations to finish for SeqSero2 in a grid computing environment, while the “sum” column represents a theoretical time if each of the SeqSero2 jobs were run sequentially one after the other.

## Discussion

**Figure 5:**
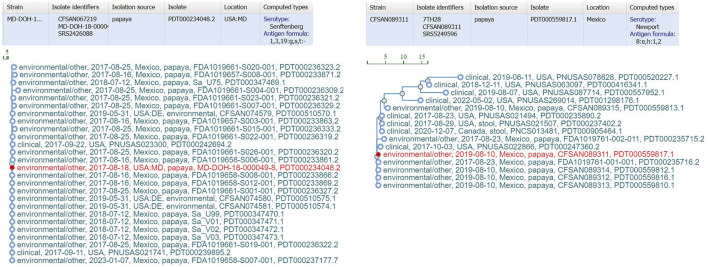
External link from the bettercallsal results table of the HTML report file to the NCBI pathogen detection website, showing SNP cluster information and computed serotype information for the papaya outbreak. The closest genome hits (red) reported by bettercallsal are the isolate genomes from the outbreak investigation for both *S*. Newport and *S*. Senftenberg according to NCBI's isolate SNP Tree Viewer.

We developed a new workflow called bettercallsal that can consistently and accurately identify *Salmonella* spp. serotypes from metagenomic or quasi-metagenomic samples. The workflow complements the abilities of the existing *in silico* protocols for *Salmonella* serotyping, such as SeqSero2, whose contributions are indirectly built into our workflows via the NCBI Pathogen Detection Project.

bettercallsal outperformed other data analysis approaches, i.e., custom *k*-mer analysis, SeqSero2, and Kallisto, for multiple serovar detection in both *in silico* and outbreak metagenomic datasets. The workflow can accommodate a wide variety of sequencing variations such as, data from a different sequencing center, different sequencing depths, etc. Furthermore, the traceback utility of bettercallsal was demonstrated in the papaya outbreak samples, where all the SNP cluster IDs for samples identified with serotypes Newport and Senftenberg clustered with the 2017 papaya outbreak isolates (Figure 5).

bettercallsal was developed for metagenomic datasets but works equally well on WGS isolate data. The advantage of bettercallsal on metagenomic data is that it works by teasing apart shared genome fractions in each sample using DNA sketching tools such as MASH and sourmash. Most of the main workflow parameters of bettercallsal are user-configurable, which can be used to accommodate the many variations of sequencing datasets and their corresponding read depths. For example, by default, up to 10 unique serotypes are retained after the MASH “screen” step (−−tuspy_n 10), which can be increased or decreased. The default parameter to filter out sequences that do not share up to a 10% coverage match with the genome hits (−−sfhpy_fcv 0.1) can also be tuned to remove these genomes from subsequent processing.

In the *in silico* datasets, *Salmonella* Sandiego (antigen formula = 4:e,h:e,n,z15) was consistently misidentified as Duisburg (antigen formula = 4:d:e,n,z15) or Nottingham (antigen formula = 16:d:e,n,z15) because of its high genomic similarity. Similarly, when the bettercallsal workflow was run on the datasets from outbreak samples, some serotypes sharing >99.99% average nucleotide identity were misidentified. For example, in the papaya outbreak enrichments from Farm A, papaya 7, the serovar Barranquilla (antigen formula = 16:d:e,n,x) was identified (Table 3; Data availability), when the call should have been Gaminara (antigen formula = 16:d:1,7). We consider Gaminara to be the correct call for several reasons. For one, the genome hit for this sample clustered with other sequences deposited as or having the “computed_serotype” of Gaminara. More technically, the top unique serovar hits for each sample from the MASH “screen” run from the bettercallsal workflow are ordered in descending order by the similarity of the reference genome “contained” in the sample FASTQ (Ondov et al., 2016). Even though the top “hit” from the screening step identifies the expected call, when we attempted to further filter down the genome hits using the genome match fraction with sourmash, we observed some misclassifications. This may be due to the highly redundant nature of the genome sequence content of *Salmonella* spp. assemblies. To date, we have only observed the serovars Gaminara and Sandiego being misclassified by bettercallsal. We are constantly working toward increasing the precision of bettercallsal by testing it on various benchmark cocktails of multiple *Salmonella* serotypes, including different variations of bioinformatics approaches.

The analysis workflows of bettercallsal and SeqSero2 each have their unique strengths. SeqSero2 is more accurate on WGS isolate datasets as it tends to identify each of the abundant O, H1, and H2 genes and computes the *Salmonella* spp. serotype based on the White-Kauffmann-Le Minor scheme (Grimont and Weill, 2007). Similarly, the Luminex xMAP assay is also only accurate for individual *Salmonella* isolates. Although multiple O and H antigens can be detected in a mixed sample, the assay does not identify which H antigens correspond to each O group present, increasing the likelihood of misidentification when multiple serotypes are present. Kallisto analysis resulted in false positives in all of these sample sets. Historically, *k-*mer analysis has been the go-to method for taxa profiling in metagenomic analyses in our laboratories. As observed in our results, the *k*-mer analysis tool was not able to detect most or all of the serovars that were part of the outbreak, and the same happened in the simulated datasets. Ultimately, in the *in silico* and real-world outbreak datasets we analyzed, bettercallsal outperformed other common methods for *Salmonella* spp. serotyping.

## Conclusion

The application of high-throughput genomic sequencing for the rapid identification and surveillance of foodborne pathogens has currently become commonplace in public health systems. We have demonstrated that shotgun metagenomic sequencing of pre-enrichment and selective enrichments (quasi-metagenomic), along with a precision analysis tool such as bettercallsal, facilitates the identification of multiple *Salmonella* serotypes and can provide traceback utility equivalent to isolate WGS.

To our knowledge, bettercallsal is one of the first analysis tools with the potential to identify multiple *Salmonella* spp. serotypes from a metagenomic or quasi-metagenomic dataset with high accuracy and can provide rapid insights into the distribution, transmission, and source tracking of a foodborne pathogen. The use of Nextflow as a workflow language enables the reproducibility of the results along with platform-agnostic process execution and an easy-to-share brief run report. Further studies are needed to ascertain confidence in the detection of the totality of *Salmonella* spp. serotypes, including the ability to work with long-read metagenomic datasets along with *de novo* genome clustering analysis.

## Data availability statement

The data presented in this study have been deposited in the NCBI database, under BioProject PRJNA952520. The download URLs for the simulated Illumina reads are provided in Supplementary Table 1. The final MultiQC HTML reports generated for the papaya and peach outbreaks discussed in this study are available at https://research.foodsafetyrisk.org/bettercallsal/manuscript/papaya_outbreak_results.html and https://research.foodsafetyrisk.org/bettercallsal/manuscript/peach_outbreak_results.html, respectively.

## Author contributions

KK conceived the workflow steps involved in bettercallsal and bettercallsal_db, designed, wrote, benchmarked, tested both the Nextflow workflows, generated the simulated datasets, and evaluated the performance of the bettercallsal workflow using precision and recall measures. PR, MM, and TK performed the *k*-mer, SeqSero2, and Kallisto analyses on the outbreak samples. AO, PR, ER, RB, KJ, RLB, CF, JZ, AW, and CG performed laboratory work on the outbreak samples. ER prepared the visuals. KK and PR wrote the manuscript. All authors revised and edited the manuscript. All authors contributed to the article and approved the submitted version.
